# Training During the COVID-19 Lockdown: Knowledge, Beliefs, and Practices of 12,526 Athletes from 142 Countries and Six Continents

**DOI:** 10.1007/s40279-021-01573-z

**Published:** 2021-10-23

**Authors:** Jad Adrian Washif, Abdulaziz Farooq, Isabel Krug, David B. Pyne, Evert Verhagen, Lee Taylor, Del P. Wong, Iñigo Mujika, Cristina Cortis, Monoem Haddad, Omid Ahmadian, Mahmood Al Jufaili, Ramzi A. Al-Horani, Abdulla Saeed Al-Mohannadi, Asma Aloui, Achraf Ammar, Fitim Arifi, Abdul Rashid Aziz, Mikhail Batuev, Christopher Martyn Beaven, Ralph Beneke, Arben Bici, Pallawi Bishnoi, Lone Bogwasi, Daniel Bok, Omar Boukhris, Daniel Boullosa, Nicola Bragazzi, Joao Brito, Roxana Paola Palacios Cartagena, Anis Chaouachi, Stephen S. Cheung, Hamdi Chtourou, Germina Cosma, Tadej Debevec, Matthew D. DeLang, Alexandre Dellal, Gürhan Dönmez, Tarak Driss, Juan David Peña Duque, Cristiano Eirale, Mohamed Elloumi, Carl Foster, Emerson Franchini, Andrea Fusco, Olivier Galy, Paul B. Gastin, Nicholas Gill, Olivier Girard, Cvita Gregov, Shona Halson, Omar Hammouda, Ivana Hanzlíková, Bahar Hassanmirzaei, Thomas Haugen, Kim Hébert-Losier, Hussein Muñoz Helú, Tomás Herrera-Valenzuela, Florentina J. Hettinga, Louis Holtzhausen, Olivier Hue, Antonio Dello Iacono, Johanna K. Ihalainen, Carl James, Dina C. Janse van Rensburg, Saju Joseph, Karim Kamoun, Mehdi Khaled, Karim Khalladi, Kwang Joon Kim, Lian-Yee Kok, Lewis MacMillan, Leonardo Jose Mataruna-Dos-Santos, Ryo Matsunaga, Shpresa Memishi, Grégoire P. Millet, Imen Moussa-Chamari, Danladi Ibrahim Musa, Hoang Minh Thuan Nguyen, Pantelis T. Nikolaidis, Adam Owen, Johnny Padulo, Jeffrey Cayaban Pagaduan, Nirmala Panagodage Perera, Jorge Pérez-Gómez, Lervasen Pillay, Arporn Popa, Avishkar Pudasaini, Alireza Rabbani, Tandiyo Rahayu, Mohamed Romdhani, Paul Salamh, Abu-Sufian Sarkar, Andy Schillinger, Stephen Seiler, Heny Setyawati, Navina Shrestha, Fatona Suraya, Montassar Tabben, Khaled Trabelsi, Axel Urhausen, Maarit Valtonen, Johanna Weber, Rodney Whiteley, Adel Zrane, Yacine Zerguini, Piotr Zmijewski, Øyvind Sandbakk, Helmi Ben Saad, Karim Chamari

**Affiliations:** 1 Sports Performance Division, Institut Sukan Negara Malaysia (National Sports Institute of Malaysia), Kuala Lumpur, Malaysia; 2 Aspetar, Orthopaedic and Sports Medicine Hospital, FIFA Medical Centre of Excellence, Doha, Qatar; 3 Melbourne School of Psychological Sciences, The University of Melbourne, Melbourne, VIC Australia; 4 Research Institute for Sport and Exercise, University of Canberra, Canberra, ACT Australia; 5 Department of Public and Occupational Health, Amsterdam Collaboration on Health & Safety in Sports, Amsterdam Movement Sciences, Amsterdam UMC, Vrije Universiteit Amsterdam, Amsterdam, The Netherlands; 6 School of Sport, Exercise and Health Sciences, National Centre for Sport and Exercise Medicine (NCSEM), Loughborough University, Loughborough, UK; 7 Human Performance Research Centre, University of Technology Sydney, Sydney, Australia; 8 Sport & Exercise Discipline Group, Faculty of Health, University of Technology Sydney, Sydney, NSW Australia; 9 School of Nursing and Health Studies, The Open University of Hong Kong, Ho Man Tin, Hong Kong; 10 Department of Physiology, Faculty of Medicine and Nursing, University of the Basque Country, Leioa, Basque Country Spain; 11 Exercise Science Laboratory, Faculty of Medicine, School of Kinesiology, Universidad Finis Terrae, Santiago, Chile; 12 Department of Human Sciences, Society and Health, University of Cassino and Lazio Meridionale, Cassino, Italy; 13 Physical Education Department, College of Education, Qatar University, Doha, Qatar; 14 Medical Committee of Tehran Football Association, Tehran, Iran; 15 Emergency Medicine Department, Sultan Qaboos University Hospital, Alkhoudh, Oman; 16 Department of Exercise Science, Yarmouk University, Irbid, Jordan; 17 World Innovation Summit for Health (WISH), Qatar Foundation, Doha, Qatar; 18 Physical Activity, Sport & Health Research Unit (UR18JS01), National Sport Observatory, Tunis, Tunisia; 19 High Institute of Sport and Physical Education, University of Gafsa, Gafsa, Tunisia; 20 Institute of Sport Sciences, Otto-Von-Guericke University, 39104 Magdeburg, Germany; 21 Interdisciplinary Laboratory in Neurosciences, Physiology and Psychology: Physical Activity, Health and Learning (LINP2), UFR STAPS, UPL, Paris Nanterre University, Nanterre, France; 22 Physical Culture, Sports and Recreation, College Universi, Pristina, Kosovo; 23 Faculty of Physical Education and Sport, University of Tetova, Tetovo, North Macedonia; 24 Sport Science and Sport Medicine, Singapore Sport Institute, Sport Singapore, Singapore, Singapore; 25 Department of Sport, Exercise and Rehabilitation, Northumbria University, Newcastle upon Tyne, UK; 26 Division of Health, Engineering, Computing and Science, Te Huataki Waiora School of Health, University of Waikato, Tauranga, New Zealand; 27 Division of Medicine, Training and Health, Institute of Sport Science and Motology, Philipps University Marburg, Marburg, Germany; 28 Applied Motion Department, Institute of Sport Research, Sports University of Tirana, Tirana, Albania; 29 Physiotherapy Department, Minerva Punjab Academy and Football Club, Mohali, Punjab India; 30 Department of Orthopedics, Nyangabgwe Hospital, Francistown, Botswana; 31 Botswana Football Association Medical Committee, Gaborone, Botswana; 32 Faculty of Kinesiology, University of Zagreb, Zagreb, Croatia; 33 High Institute of Sport and Physical Education, University of Sfax, Sfax, Tunisia; 34 INISA, Federal University of Mato Grosso do Sul, Campo Grande, Brazil; 35 Sport and Exercise Science, James Cook University, Townsville, QLD Australia; 36 Laboratory for Industrial and Applied Mathematics (LIAM), Department of Mathematics and Statistics, York University, Toronto, ON M3J 1P3 Canada; 37 Portugal Football School, Portuguese Football Federation, Oeiras, Portugal; 38 Facultad de Ciencias del Deporte, Universidad de Extremadura, Cáceres, Spain; 39 Tunisian Research Laboratory, Sport Performance Optimisation, National Center of Medicine and Science in Sports (CNMSS), Tunis, Tunisia; 40 Sports Performance Research Institute New Zealand, AUT University, Auckland, New Zealand; 41 Department of Kinesiology, Brock University, St. Catharines, ON Canada; 42 Faculty of Physical Education and Sport, University of Craiova, Craiova, Romania; 43 Faculty of Sport, University of Ljubljana, Ljubljana, Slovenia; 44 Department of Automation, Biocybernetics and Robotics, Jozef Stefan Institute, Ljubljana, Slovenia; 45 Right to Dream Academy, Old Akrade, Ghana; 46 Sport Science and Research Department, Centre Orthopédique Santy, FIFA Medical Centre of Excellence, Lyon, France; 47 Laboratoire Interuniversitaire de Biologie de la Motricité (LIBM EA 7424), Claude Bernard University (Lyon 1), Lyon, France; 48 Department of Sports Medicine, Hacettepe University, Ankara, Turkey; 49 Al Hilal Football Club, Riyadh, Saudi Arabia; 50 Paris Saint Germain FC, Paris, France; 51 Health and Physical Education Department, Prince Sultan University, Riyadh, Kingdom of Saudi Arabia; 52 Department of Exercise and Sport Science, University of Wisconsin-La Crosse, La Crosse, WI USA; 53 Sport Department, School of Physical Education and Sport, University of São Paulo, São Paulo, Brazil; 54 Interdisciplinary Laboratory for Research in Education, EA 7483, University of New Caledonia, Avenue James Cook, 98800 Nouméa, New Caledonia; 55 Sport and Exercise Science, School of Allied Health, Human Services and Sport, La Trobe University, Melbourne, VIC Australia; 56 New Zealand All Blacks, New Zealand Rugby, Wellington, New Zealand; 57 School of Human Science (Exercise and Sport Science), The University of Western Australia, Perth, WA Australia; 58 School of Behavioural and Health Sciences, McAuley at Banyo, Australian Catholic University, Brisbane, QLD Australia; 59 Interdisciplinary Laboratory in Neurosciences, Physiology and Psychology: Physical Activity, Health and Learning (LINP2), UPL, UFR STAPS, Paris Nanterre University, Nanterre, France; 60 Research Laboratory, Molecular Bases of Human Pathology, Faculty of Medicine, LR19ES13, University of Sfax, Sfax, Tunisia; 61 Sports Medicine Research Center, Neuroscience Institute, Tehran University of Medical Sciences, Tehran, Iran; 62 Iran Football Medical Assessments and Rehabilitation Center, IFMARC, Tehran, Iran; 63 School of Health Sciences, Kristiania University College, Oslo, Norway; 64 Department of Economic-Administrative Sciences, Universidad Autónoma de Occidente, Los Mochis, Sinaloa México; 65 Department of Sport Science and Health, Universidad Santo Tomás, Santiago, Chile; 66 University of Santiago of Chile (USACH), Sciences of Physical Activity, Sports and Health School, Santiago, Chile; 67 Weil-Cornell Medical College in Qatar, Doha, Qatar; 68 Section Sports Medicine, Faculty of Health Sciences, University of Pretoria, Pretoria, South Africa; 69 Department of Exercise and Sports Science, University of the Free State, Bloemfontein, South Africa; 70 Laboratoire ACTES, UFR-STAPS, Université Des Antilles, Pointe à Pitre, France; 71 School of Health and Life Sciences, University of the West of Scotland, Hamilton, UK; 72 Faculty of Sport and Health Sciences, Biology of Physical Activity, University of Jyväskylä, Jyväskylä, Finland; 73 Medical Board Member, International Netball Federation, Manchester, UK; 74 High Performance Director, Sports Authority of India, Bangalore, India; 75 SEHA, Singapore, Singapore; 76 Department of Internal Medicine, Yonsei University College of Medicine, Seoul, South Korea; 77 Department of Sport Science, Tunku Abdul Rahman University College, Kuala Lumpur, Malaysia; 78 Sport Science Department, Fulham Football Club, Fulham, London, UK; 79 Centre for Trust, Peace and Social Relation, Coventry University, Coventry, UK; 80 Department of Sport Management, Faculty of Management, Canadian University of Dubai, Dubai, United Arab Emirates; 81 Programa Avancado de Cultura Contemporanea, Universidade Federal Do Rio de Janeiro, Rio de Janeiro, Brazil; 82 Antlers Sports Clinic, Kashima, Ibaraki Japan; 83 Department of Orthopedic Surgery, Tokyo Medical University, Tokyo, Japan; 84 Faculty of Physical Education, University of Tetovo, Tetovo, North Macedonia; 85 Institute of Sport Sciences, University of Lausanne, Lausanne, Switzerland; 86 Department of Human Kinetics and Health Education, Kogi State University, Anyigba, Nigeria; 87 Ho Chi Minh City University of Sport, Ho Chi Minh, Vietnam; 88 School of Health and Caring Sciences, University of West Attica, Attica, Greece; 89 University Claude Bernard Lyon 1, Lyon, France; 90 Seattle Sounders Football Club, Seattle, WA USA; 91 Department of Biomedical Sciences for Health, Università Degli Studi di Milano, Milan, Italy; 92 School of Health Sciences, College of Health and Medicine, University of Tasmania, Launceston, TAS Australia; 93 Sports Medicine, Australian Institute of Sport, Bruce, ACT Australia; 94 University of Canberra Research Institute for Sport and Exercise (UCRISE), University of Canberra, Bruce, ACT Australia; 95 Nuffield Department of Orthopaedics, Rheumatology and Musculoskeletal Sciences, University of Oxford, Oxford, UK; 96 Health, Economy, Motricity and Education (HEME) Research Group, Faculty of Sport Sciences, University of Extremadura, Cáceres, Spain; 97 University of Witwatersrand, Wits Institute for Sports Health, Johannesburg, South Africa; 98 Health and Sport Science Department, Educational Faculty, Mahasarakham University, Mahasarakham, Thailand; 99 Medical Department, All Nepal Football Association (ANFA), Lalitpur, Nepal; 100 Department of Exercise Physiology, College of Sport Sciences, University of Isfahan, Isfahan, Iran; 101 Faculty of Sport Science, Universitas Negeri Semarang, Semarang, Indonesia; 102 Krannert School of Physical Therapy, University of Indianapolis, Indianapolis, IN USA; 103 Bashundhara Kings, Nilphamari, Bangladesh; 104 Miskawaan Health Group, Bangkok, Thailand; 105 Department of Sports Science and Physical Education, University of Agder, Kristiansand, Norway; 106 Physiotherapy Department, BP Eyes Foundation CHEERS Hospital, Bhaktapur, Nepal; 107 Research Laboratory: Education, Motricity, Sport and Health, EM2S, LR19JS01, University of Sfax, Sfax, Tunisia; 108 Sports Clinic, Centre Hospitalier de Luxembourg, Clinique d‘Eich, Luxembourg, Luxembourg; 109 Luxembourg Institute of Research in Orthopedics, Sports Medicine and Science, Luxembourg, Luxembourg; 110 Human Motion, Orthopedics, Sports Medicine and Digital Methods, Luxembourg Institute of Health, Luxembourg, Luxembourg; 111 Research Institute for Olympic Sports, Jyvaskyla, Finland; 112 Institute for Sports Science, CAU of Kiel, Kiel, Germany; 113 Neurocognition and Action, University of Bielefeld, Bielefeld, Germany; 114 University of Queensland, Brisbane, QLD Australia; 115 Department of Physiology and Lung Function Testing, Faculty of Medicine of Sousse, University of Sousse, Sousse, Tunisia; 116 Faculty of Sciences of Bizerte, University of Carthage, Bizerte, Tunisia; 117 High Institute of Sports, Ksar Said, Tunis, Tunisia; 118 FIFA Medical Centre of Excellence Algiers, Algiers, Algeria; 119 Medical Committee, Confederation of African Football, Giza, Egypt; 120 Jozef Pilsudski University of Physical Education in Warsaw, Warsaw, Poland; 121 Centre for Elite Sports Research, Department of Neuromedicine and Movement Science, Norwegian, University of Science and Technology, Trondheim, Norway; 122 Laboratoire de Recherche “Insuffisance Cardiaque” (LR12SP09), Hôpital Farhat HACHED, Université de Sousse, Sousse, Tunisie; 123 Laboratoire de Physiologie, Faculté de Médicine de Sousse, Université de Sousse, Sousse, Tunisie

## Abstract

**Objective:**

Our objective was to explore the training-related knowledge, beliefs, and practices of athletes and the influence of lockdowns in response to the coronavirus disease 2019 (COVID-19) pandemic caused by severe acute respiratory syndrome coronavirus 2 (SARS-CoV-2).

**Methods:**

Athletes (*n* = 12,526, comprising 13% world class, 21% international, 36% national, 24% state, and 6% recreational) completed an online survey that was available from 17 May to 5 July 2020 and explored their training behaviors (training knowledge, beliefs/attitudes, and practices), including specific questions on their training intensity, frequency, and session duration before and during lockdown (March–June 2020).

**Results:**

Overall, 85% of athletes wanted to “maintain training,” and 79% disagreed with the statement that it is “okay to not train during lockdown,” with a greater prevalence for both in higher-level athletes. In total, 60% of athletes considered “coaching by correspondence (remote coaching)” to be sufficient (highest amongst world-class athletes). During lockdown, < 40% were able to maintain sport-specific training (e.g., long endurance [39%], interval training [35%], weightlifting [33%], plyometric exercise [30%]) at pre-lockdown levels (higher among world-class, international, and national athletes), with most (83%) training for “general fitness and health maintenance” during lockdown. Athletes trained alone (80%) and focused on bodyweight (65%) and cardiovascular (59%) exercise/training during lockdown. Compared with before lockdown, most athletes reported reduced training frequency (from between five and seven sessions per week to four or fewer), shorter training sessions (from ≥ 60 to < 60 min), and lower sport-specific intensity (~ 38% reduction), irrespective of athlete classification.

**Conclusions:**

COVID-19-related lockdowns saw marked reductions in athletic training specificity, intensity, frequency, and duration, with notable within-sample differences (by athlete classification). Higher classification athletes had the strongest desire to “maintain” training and the greatest opposition to “not training” during lockdowns. These higher classification athletes retained training specificity to a greater degree than others, probably because of preferential access to limited training resources. More higher classification athletes considered “coaching by correspondence” as sufficient than did lower classification athletes. These lockdown-mediated changes in training were not conducive to maintenance or progression of athletes’ physical capacities and were also likely detrimental to athletes’ mental health. These data can be used by policy makers, athletes, and their multidisciplinary teams to modulate their practice, with a degree of individualization, in the current and continued pandemic-related scenario. Furthermore, the data may drive training-related educational resources for athletes and their multidisciplinary teams. Such upskilling would provide athletes with evidence to inform their training modifications in response to germane situations (e.g., COVID related, injury, and illness).

**Supplementary Information:**

The online version contains supplementary material available at 10.1007/s40279-021-01573-z.

## Key Points

**Table:** 

Higher classification athletes have superior knowledge and beliefs/attitudes regarding training, although these were ranked predominately as “moderate,” suggesting that training-related evidence may not penetrate all athletes to a “good” level.
During lockdown, most athletes trained alone and focused on general health and well-being rather than with sport or discipline specificity, partly because of a lack of resource such as space, equipment, facilities, and multidisciplinary support teams, with such access favoring higher classification athletes.
The challenges athletes experienced during lockdown reduced their motivation, which was amplified by the lack of competition. Athletes/coaches may benefit from arrangements that permit training and competition during lockdown (even if home based).
Although higher classification athletes coped better in general, all athletes reported substantial reductions in key training variables, including frequency, duration, intensity, and type.
“Remote”-based practices using digitally mediated technology for coaching/training emerged, appeared effective, and were best received by higher classification athletes.
Information resources (e.g., easily accessible online seminars and discussions) are necessary for athletes to improve knowledge and beliefs/attitudes.

## Introduction

The coronavirus disease 2019 (COVID-19) pandemic caused by severe acute respiratory syndrome coronavirus 2 (SARS-CoV-2) compromised the ability of many populations to engage in physical activity and benefit from sport participation [1]. Both recreational and elite competition schedules were decimated by postponements and cancellations, including the 2020 Tokyo Olympic Games. Athlete training was compromised for numerous reasons, most crucially the periods of recurring local/national lockdowns (including movement restrictions, social distancing, and facility closures). Closures of specialist athlete training facilities were widespread, hindering athlete access to these and their multidisciplinary teams (e.g., coaches, sports science, medical and allied health professionals) [2, 3]. Team or contact sports have been particularly challenged because social distancing prevents physical interaction and much team-based technical and tactical training [4]. In combination, these challenges have compromised the ability of high-performance athletes to conduct their physical, technical, or tactical training [5].

To comply with lockdown restrictions, many creative—often home-based—training solutions were employed in attempts to facilitate appropriate training load, maintain/progress physical and technical qualities, and minimize injury risk [2, 5–8]. Performing these exercises during lockdown could also boost immunity and anti-inflammatory effects (reduced risk of disease) in response to respiratory pathogens such as seasonal influenza [9]. This pandemic-associated lockdown could have negative physical consequences, including reduced maximal oxygen consumption, endurance capacity, muscular strength, and muscle mass [10]. Mental health can also be adversely affected by the stress or anxiety experienced in isolation during lockdown [11]. Limited data regarding altered athlete training practice in response to lockdown have emerged, albeit specific to one country (South Africa) experiencing a high COVID-19 burden [5]. Here, athletes of different classifications (elite and subelite) reported training at altered moderate intensities for reduced session lengths during lockdown [5]. Substantial reductions in weekly training frequency and time were reported among collegiate-level athletes from different sports [12]. More recently, a worldwide study within handball reported reduced physical activity and increased sedentary behavior, regardless of the athlete’s competitive level [13].

Some general guidelines for physical activity during lockdown have been suggested [14], although these indirectly touched upon exercise without empirical data on athletes’ training practices. Other recommendations were largely generic and likely insufficient for different levels of athletes, such as state and world-class levels [15, 16]. During early phases of the pandemic, “return to sport” considerations were focused on higher-level athletes [3, 17]. These studies provided useful insights related to the safety of training and competition during the pandemic but fell short of evidence-based guidelines for athletes across all competitive levels.

Athletes’ experience may be conceptualized as the extent to which they engage in exercise, training, and competition [18]. During lockdown, athletes may be more dependent on themselves, instead of on their coach, which further supports the importance of self-regulation ability (e.g., metacognitively, motivations, and actions) [19]. In this context, self-efficacy may be thought of as a motivational mechanism for self-regulated learners, which refers to a person’s beliefs in their abilities to think and act in ways that progress them towards their learning goals [20]. Additionally, personal beliefs could act as a placebo that impacts on training routines [21]. Among adults, knowledge of both aerobic and muscular types of physical activity recommendations was positively associated with physical fitness variables [22]. Similarly, positive attitudes were associated with being physically active [23], whereas having positive attitudes and beliefs about exercise or being physically active for health predicted physical activity participation [24]. Likewise, athletes’ concerns over reduced fitness and abilities could have influenced their attitudes toward training during lockdown. Based on these premises, an athlete’s ability to successfully react to the training-related challenges of COVID-19 and modify their practice (e.g., training intensity, volume, frequency, and mode) could be influenced by their existing knowledge of and beliefs or attitudes about training. Thus, identifying the knowledge, beliefs, and attitudes of athletes related to training and training interruptions (including COVID-19), while accounting for athlete classification, their adaptive responses to training (i.e., those with higher self-regulatory skills would train more), and degree of remote coaching and practices, is warranted.

Globally derived data from a variety of athletes (world class or otherwise) are required to elucidate the effects of lockdown on their training practices. Such evidence may help policy makers, the athletes, and their multidisciplinary teams modulate their practice, with a degree of individualization, in the current and continued COVID-related scenario [25]. Understanding how public health measures influence athletes may help better prepare sports medicine and support teams for similar situations in the future. For these reasons, we characterized the athletes’ knowledge and beliefs/attitudes related to training disruptions and practices during the COVID-19 lockdown in a large global sample, including comparisons between athlete classifications (e.g., world-class, national, and state-level athletes).

## Methods

### Design and Participants

Participants provided informed consent, and the study received ethical approvals from the University of Melbourne Human Research Ethics Committee (HREC; no. 2056955.1), Qatar University (QU-IRB 1346-EA/20), and the University of Cassino e Lazio Meridionale (10031) in the spirit of the Declaration of Helsinki. Data were collected and processed anonymously and according to the guidelines of the “General Data Protection Regulation” (gdpr-info.eu). Participation was voluntary, and all individuals were permitted to withdraw at any time before completion and submission of the survey. Participant eligibility criteria were as follows: (1) elite- or subelite athletes aged ≥ 18 years of either sex with or without disability; (2) athletes experienced at least two consecutive weeks of lockdown (March–June 2020); (3) athletes had not missed training for ≤ 7 days because of illness/injury within the survey period; and (4) athletes experienced a “medium-to-high” lockdown severity. A medium–high lockdown severity was considered met when one or more of the following criteria were fulfilled: (1) movement was permitted only for essential supplies and groceries, (2) access to public exercise facilities was restricted (i.e., recreational areas such as parks or open spaces were closed or time/capacity limits were imposed), and (3) training facilities at institutions, clubs, colleges, etc. were closed. The a priori sample size estimation was 12,418 (see the supplementary material S1). In total, 13,772 entries were evident upon survey closure. After exclusions (*n* = 1246) for duplicates (*n* = 731), age limit violations (*n* = 410), and/or unmet lockdown severity criteria (*n* = 105) were completed, a final sample of data from 12,526 athletes (142 countries/territories across six continents) was used for subsequent statistical modeling. The sample represented 108 “team” and “individual” sports.

### Protocols and Questionnaires

#### Data Collection

An online survey was administered and disseminated via Google Forms from 17 May to 5 July 2020. The survey was shared via email and personal/group messaging applications (e.g., WhatsApp, Signal, and Telegram) and promoted on social media (e.g., Facebook, Twitter, and Instagram) through the professional networks of the research team (e.g., clubs, federations, and institutions). The English language “master” version of the survey was translated and administered in 34 further languages: Albanian, Arabic, Bangla, Chinese-simplified, Chinese-traditional, Croatian, Czech, Danish, Finnish, French, German, Greek, Hindi, Indonesian, Italian, Japanese, Korean, Malay, Nepalese, Norwegian, Persian, Polish, Portuguese, Punjabi, Romanian, Russian, Sinhala, Slovenian, Spanish, Swahili, Swedish, Thai, Turkish, and Vietnamese. The survey questions underwent translation and back-translation, performed by the research team (including at least one native speaker and one topic expert), including pilot completions of the survey by and feedback from native language speaking athletes, resulting in the finalized surveys for all languages.

Data from questions with preset answers (i.e., predefined multiple choice) were converted directly into standardized codes/numbers using an automated/customized setting on the Excel spreadsheet (Microsoft Corporation; Redmond, WA, USA); all automated responses were checked for veracity. Remaining data (i.e., free-text answers) underwent theme analysis/aggregation (all non-English responses were back-translated to English first), and subsequent themes were re-classified into standardized codes/numbers to facilitate statistical modeling. Test–retest reliability was determined within an English-speaking participant subgroup (*n* = 129), under the same conditions, twice (separated by 9 ± 4 days), with Cronbach’s alpha (0.82–0.97) rated as good to excellent [26].

#### Survey Questionnaire

The survey was initially developed by the first and senior author and then reviewed by the wider authorship team (e.g., research team), involving > 100 researchers (from > 60 countries). The 59 questions explored athletes’ training knowledge, beliefs/attitudes, and practices, including specific questions (intensity, frequency, and session duration) on their training before and during lockdown, within a structure of four sections (see the ESM in conjunction with the following text/section (Sect.) for specific questions in the present study).

- Athlete details (11 questions): Athlete classification: (1) Olympic Games, world championships, or equivalent (categorized as world class); (2) other international events (international); (3) national; (4) state or province (state); and (5) others (recreational).
- Athlete knowledge (ten questions). Athletes’ views (what was known) on training disruptions during lockdown and its associated effects were assessed. A 5-point Likert scale (1 = “strongly agree” to 5 = “strongly disagree” and 6 = “don’t know”) established (1) general training knowledge (e.g., training volume and intensity required to maintain fitness); and (2) how athletes attempted to continue their training during lockdown and thus how lockdown affected their training.
- Athletes’ beliefs and attitudes (14 questions): How the athletes perceived training interruptions during lockdown and their implications for training. Specifically, the athletes expressed what they thought or believed and how they behaved optimistically (attitude) towards key issues. The same 5-point Likert scale explored athletes’ perceptions of fitness, mental health and emotion, coaching interaction, desire to train, and motivation.
- Training practices (ten questions): An array of question styles was used to establish training practices, including (1) selecting one or more predefined answers; (2) comparing related before and during lockdown effects on training practices; (3) yes or no; and (4) sub-questions including a free-text cell to capture nuanced detail.

A scoring system was developed where knowledge (Sect. b) had nine scored questions (scoring range: 0–9) and beliefs/attitudes (Sect. c) had seven scored questions (scoring range: 0–7). Correct (for knowledge) or positive (for beliefs/attitudes) answers (e.g., strongly agree/agree or strongly disagree/disagree with a statement) were scored as “1.” The other answers received a score of “0” (including the statements “neutral” or “don’t know”). The total score was used to rank the level of knowledge and beliefs/attitudes (i.e., ≥ 70% as good, 51 to ˂ 70% as moderate, and ≤ 50% as poor), as used previously [27, 28] to compare athletes of different classifications.

### Statistical Analysis

All data were coded and statistical analyses performed using SPSS v. 23 (IBM; Armonk, NY, USA). Data are presented using a variety of appropriate descriptive statistics, including frequencies, percentages, and mean ± standard deviation. Knowledge and belief/attitude scores between athlete classifications were modeled using a one-way analysis of variation and effect size (*η*^2^) with a Bonferroni analysis post hoc if indicated. The chi-squared test was used to compare categorical variables between athlete classifications. Adjusted standardized residuals from the chi-squared tests were interpreted to determine significant associations. Pearson’s correlation coefficient (*r*) analysis was used to examine the associations between knowledge and belief/attitude scores, and between knowledge and training variables (frequency, duration, and intensity) and belief/attitude and training variables. Two-tailed alpha was < 0.05.

## Results

### Demographic

Table 1 shows the demographic characteristics of athletes. Athletes were predominantly men (66%), aged 18–29 years (67%), from 108 sports. Most (83%) had experienced lockdown for 5–12 weeks at survey completion, with two-thirds (67%) permitted to exercise only at home (Table 2).

**Table 1:** Demographic characteristics of participants (*n* = 12,526)

Characteristics	Number (%)
Sex	
Male	8265 (66)
Female	4229 (34)
Other	32 (0)
Age category, years	
18–29	8419 (67)
30–39	2431 (19)
40–49	1078 (9)
50–59	468 (4)
≥ 60	121 (1)
Missing	9 (−)
Continent	
Asia	4777 (38)
Europe	4305 (34)
Africa	1375 (11)
South America	973 (8)
North America	907 (7)
Oceania	189 (2)
Athlete’s status	
Amateur	6453 (51)
Semiprofessional	2765 (22)
Professional	3222 (26)
Other	86 (1)
Main sports	
Soccer	2696 (22)
Athletics	1306 (10)
Cycling	679 (5)
Volleyball	602 (5)
Basketball	522 (4)
Triathlon	503 (4)
Handball	403 (3)
Rugby	365 (3)
Swimming	348 (3)
Judo	313 (3)
Taekwondo	254 (2)
Hockey	210 (2)
Futsal	198 (2)
Karate	165 (1)
Baseball/Softball	159 (1)
Netball	145 (1)
Rowing	130 (1)
Bodybuilding	130 (1)
Cricket	124 (1)
Fencing	121 (1)
Other sports	3153 (25)
Sports experience, years	
≤ 3	1476 (12)
4–9	4191 (34)
10–19	5055 (41)
≥ 20	1645 (13)
Missing	159 (−)
Athlete classification	
World class	1674 (13)
International	2565 (21)
National	4482 (36)
State	3038 (24)
Recreational	763 (6)
Missing	4 (−)
Are you currently in lockdown?	
Yes	7955 (64)
No	4568 (36)
Missing	3 (−)
Lockdown experience, weeks	
≤ 4	1809 (15)
5–8	4256 (35)
9–12	5839 (48)
≥ 12	278 (2)
Missing	344 (−)
Number of household members	
1 (live alone)	815 (7)
2	2012 (16)
3	2468 (20)
4	3376 (27)
≥ 5	3767 (30)
Missing	88 (−)

**Table 2:** Training and exercise during lockdown (*n* = 12,526)

What the governing authority allowed during lockdown	Number (%)
Exercising at home only	8330 (67)
Using available spaces for exercise around my housing area/compound	5256 (42)
Outdoor cycling	3354 (27)
Running in a recreational park or stadium	3317 (27)
Outdoor hiking or trekking in non-public facilities	2577 (21)
Receive/borrow equipment from sports bodies or institutes and train at home	2105 (17)
Access to gymnasium (muscle strengthening/resistance training)	579 (5)
Access to sports academy or institute’s school or university’s facilities	510 (4)
Other	100 (1)

As athletes could select multiple answers for all questions, the numbers do not total 12,526 or 100%

### Knowledge and Beliefs/Attitudes

The results for questions related to knowledge (S2) and beliefs/attitudes (S3) used for the summed scores (interpretive thresholds [e.g., moderate] described previously) are presented in the ESM. Summed knowledge and belief/attitude scores related to training interruptions were 57 and 55%, respectively (considered moderate), with generally higher scores in higher classification athletes (*p* < 0.05) (Table 3).

**Table 3:** Comparison of knowledge and beliefs/attitudes related to training interruptions during lockdown among athlete classification from world class to recreational (*n* = 12,495)

Classification	Knowledge (range 0–9 marks)	Beliefs/attitudes (range 0–7 marks)
World class	5.2 ± 1.6 (58%)	3.9 ± 1.5 (56%)
International	5.2 ± 1.6 (58%)	3.9 ± 1.6 (56%)
National	5.1 ± 1.7 (57%)	3.8 ± 1.6 (54%)
State	5.1 ± 1.6 (57%)	3.9 ± 1.6 (56%)
Recreational	4.8 ± 1.8 (53%)*	3.4 ± 1.7 (49%)*
Total	5.1 ± 1.7 (57%)	3.8 ± 1.6 (54%)
Effect size	0.003	0.005

Higher scores indicate a greater number of correct (for knowledge) or positive (for beliefs/attitudes) answers (e.g., strongly agree/agree or strongly disagree/disagree with a statement); Data are mean ± standard deviation

^*^Significantly different from all other athlete classifications

Athlete classification was positively associated with knowledge (*p* < 0.05), except for “training frequency” (*p* = 0.073) (S4, question 4 [Q4]). Athletes (particularly national athletes) “agreed” (39%) or “strongly agreed” (29%) that lockdown limited training and potentially reduced fitness (Q1), but athletes “disagreed” (37%) or “strongly disagreed” (25%; particularly “world-class” athletes [*p* < 0.05]) that “normal” training was possible during lockdown (Q7), see S4. Many athletes (60%, with the highest among world-class athletes) considered “coaching by correspondence (remote coaching)” to be sufficient.

Additionally, knowledge was positively correlated with beliefs/attitudes (*r* = 0.41), but there was little association with training frequency (*r* =  − 0.03), duration (*r* =  − 0.06), or intensity (*r* =  − 0.08). Similarly, belief/attitude scores were not related to self-reported training frequency (*r* = 0.05), duration (*r* =  − 0.002), or intensity (*r* =  − 0.01).

### Training Practices

**Fig. 1:**
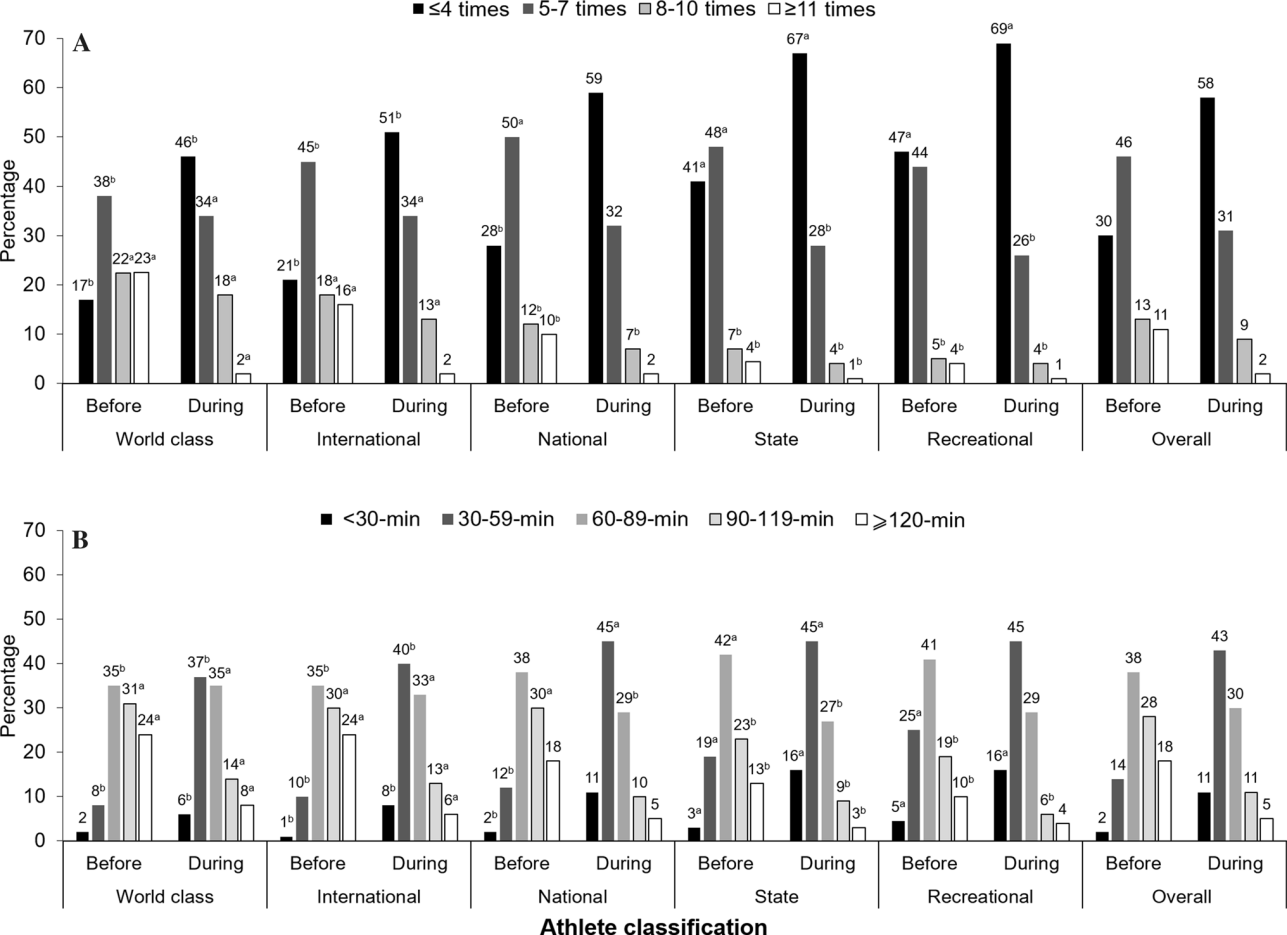
Training frequency and duration. **A** Your frequency of training sessions per week (representative of most of lockdown)? (*n* = 11,646). **B** How long do/did you train during each training session? (*n* = 10,147). For both training “frequency” and “duration” a significant relationship (*χ*^2^) existed with the athlete classification both “before” and “during” the lockdown *p* < 0.001%, within athlete classification, represent “yes” answer, relative to “no” answer. ^a^Significantly higher; ^b^Significantly lower. *before* indicates before lockdown, *during* indicates during lockdown

**Fig. 2:**
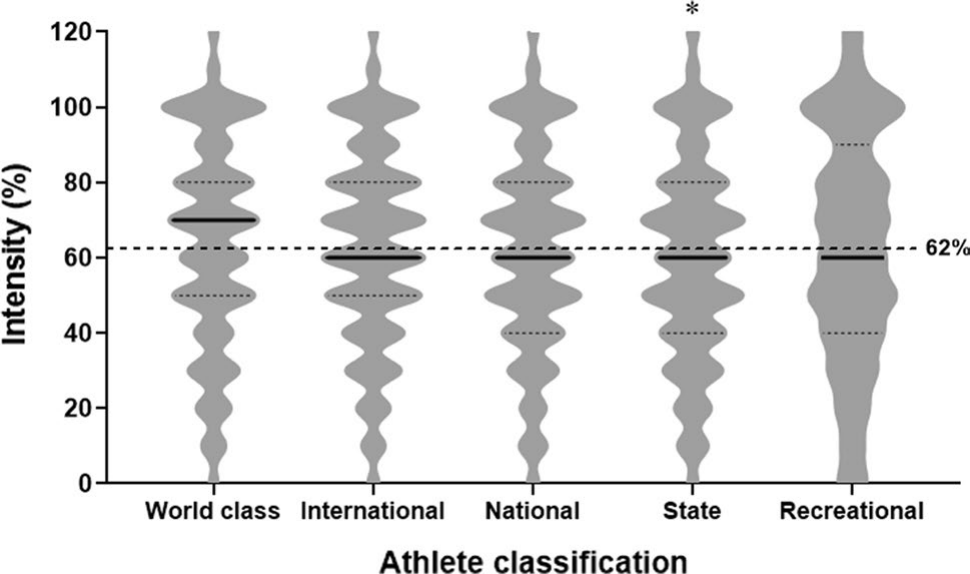
Training intensity during lockdown. Question: Do/did you maintain your pre-lockdown intensity for sports-specific training (practicing your sport) during the lockdown? Can you estimate how much in percentage? (100% represents the same intensity as before the lockdown) (*N* = 12,518). The dotted line represents average intensity across athlete classification (62%). *Significant difference from world class, international, and national. The violin plot includes a 5-point summary (lowest to highest): minimum, first quartile, median, third quartile, and maximum. The maximum or minimum number in the dataset, respectively, is shown by the upper extreme or lower extreme of the chart. Upper (third, dotted line) and lower (first; dotted line) quartiles, respectively are the 75th and 25th percentiles. The median (middle of data set) is shown as a line (i.e., thicker) in the center of each chart

**Fig. 3:**
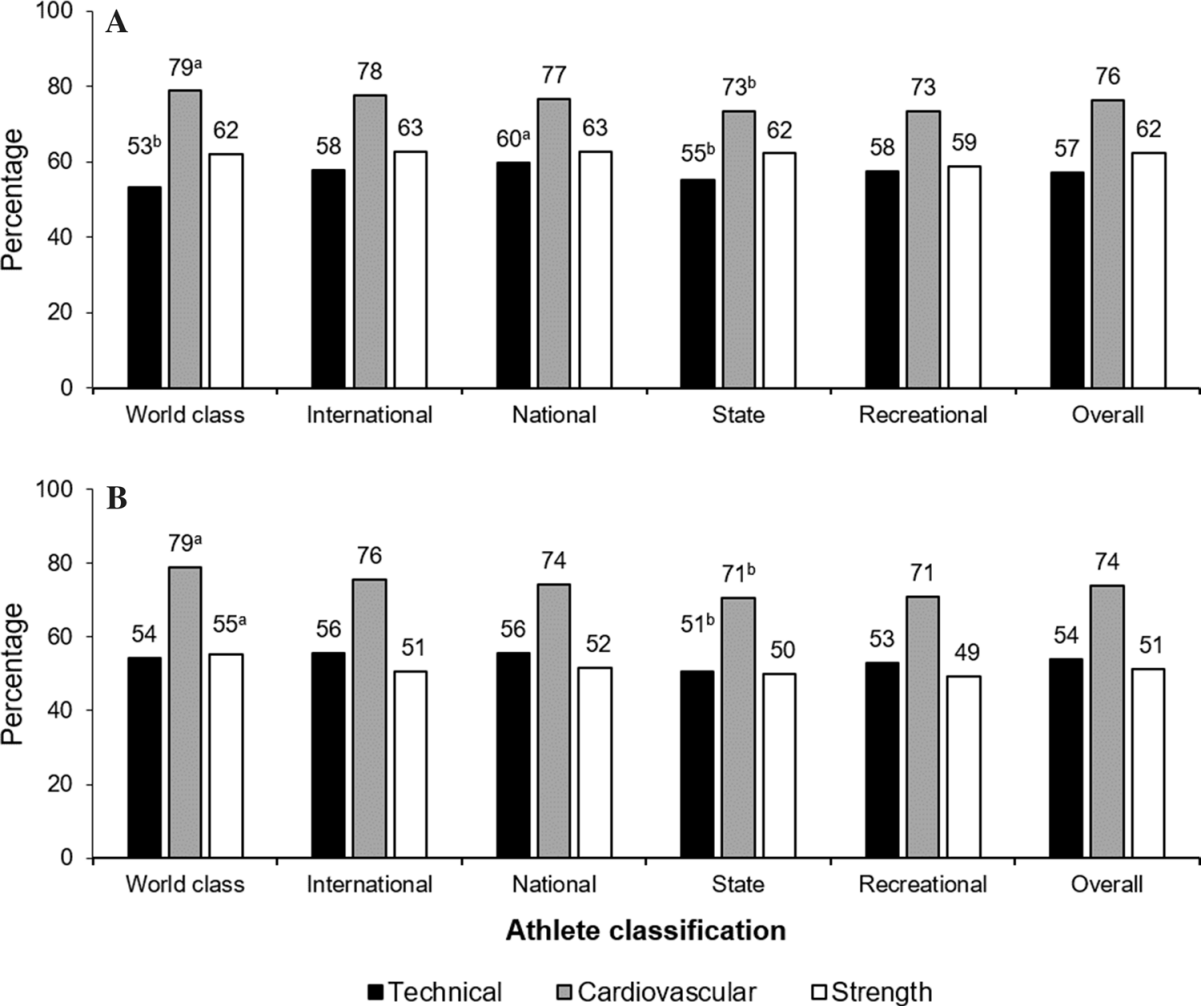
Reported practices for space/access and equipment to training (*n* = 11,451). Do/did you have **A** sufficient space/access and **B** necessary equipment to train. Significance of relationship indicated by the chi-squared test for independence. %, within athlete classification, represents a “yes” answer relative to a “no” answer. *Significant relationship with athlete classification, *p* < 0.05. ^a^Significantly higher. ^b^Significantly lower. Technical skills training: “cardiovascular” consisted of running, cycling, jogging, and high-intensity interval training, “strength” consisted of weightlifting training

Most questions were positively related to athlete classification (*p* < 0.05). During lockdown, 83% of athletes aimed to maintain or develop general fitness and health, generally (80%) training alone (particularly world-class athletes [*p* < 0.05]). Many athletes (65%), especially world-class athletes (*p* < 0.05) used bodyweight-based exercises with limited or repurposed equipment/items. Only < 40% managed to perform specific training (e.g., long endurance and interval training) at an intensity similar to that before lockdown (more so in higher classification athletes) (Table 4). Compared with before lockdown, training frequency was reduced from between five and seven sessions per week to four or fewer during lockdown (Fig. 1a), with ~ 70% (before lockdown) to ~ 42% (during lockdown) of athletes training for five or more sessions per week. A longer (≥ 60 min) to shorter (< 60 min during lockdown) training duration per session was evident (Fig. 1b), although more world-class and international athletes trained for at least 90-min periods before and during lockdown. Proportions of athletes who trained ≥ 60 min per session were higher (~ 84%) before than during (~ 46%) lockdown. Training intensity was reduced (~ 38%) on average, with state athletes reporting lower training intensity (*p* < 0.05) than other athlete classifications (Fig. 2). Access to space and equipment (to facilitate technical, cardiovascular, and strength training) was related to athlete classification (*p* < 0.001), aside from strength training space/equipment (*p* = 0.018) (Fig. 3).

**Table 4:** Athlete practices during COVID-19 lockdown

Practice	Percentage					
	WC	INT	NAT	ST	REC	Overall
1. What are/were your general purpose(s) of training during the lockdown? (*n* = 12,385)						
To maintain/develop general fitness/health*	84	83	81^b^	83	84	83
To maintain/develop skills/technique*	44	44	44^a^	40^b^	33^b^	43
To maintain/develop strength and power*	56	58^a^	55	52^b^	45^b^	54
To maintain/develop muscular endurance*	57	58^a^	55	52^b^	49^b^	55
To maintain/develop abdominal strength*	50	52^a^	50^a^	43^b^	40^b^	48
To maintain/develop aerobic fitness*	50	53^a^	51	46^b^	46^b^	50
To maintain/develop general flexibility*	49^a^	49^a^	43	38^b^	39^b^	44
To improve muscle balance*	39^a^	40^a^	36	34^b^	31^b^	36
Weight management*	46	48	47	47	54^a^	48
Other*	1	1	1^b^	1^a^	2^a^	1
2. Who is prescribing/prescribed the training program during the lockdown? (*n* = 12,351)						
Own training program*	35^b^	34^b^	42	54^a^	54^a^	44
Training program from my coach/trainer*	46^a^	45^a^	42^a^	30^b^	30^b^	40
Combined own training and coach/trainer*	44^a^	44^a^	37	29^b^	23^b^	36
Found training material from an external source: online/social media/TV, a friend, etc.*	20^b^	25	24^b^	30^a^	30^a^	26
Other*	0	0	0	1	2^a^	0
3. Do/did you train? (*n* = 12,347)						
Alone*	82^a^	78^b^	78^b^	81	82	80
In a small group of partners of equal athletic capacity*	31	32^a^	31^a^	25^b^	21^b^	29
With family members or friends with little athletic capacity*	22^a^	19	18	18	18	19
Other	1	1^a^	1	1^b^	1	1
4. What are the type of exercises that you are doing/have been doing consistently (at least twice a week) during lockdown? (*n* = 12,522)						
Bodyweight-based exercises with limited equipment*	68^a^	66	64	64	56^b^	65
Weightlifting/strength training with suitable equipment (dumbbells, weights, etc.)*	40^a^	34^a^	31^b^	29^b^	26^b^	32
Technical skills (sport-specific skills)*	41^a^	40^a^	37	33^b^	28^b^	36
Imitation or simulation of the techniques of my sport*	30^a^	27^a^	25	20^b^	21^b^	25
Cardiovascular training (running, cycling, jogging, rowing), including HIIT*	65^a^	62^a^	59	56^b^	51^b^	59
Plyometric training (repeated jumping)	26	31^a^	28^a^	20^b^	15^b^	26
Other*	1	1	1^b^	1	4^a^	1
5. What are the types of specific training you are/were able to do with the same intensity during the lockdown (very similar to pre-lockdown)? (*n* = 12,522)						
Warm-up and stretching*	84^a^	82	81	80^b^	78^b^	81
Weightlifting (strength) training*	35	32	33	34	27^b^	33
Plyometric training (e.g., repeated jumping)*	31	34^a^	32^a^	25^b^	20^b^	30
Technical skills (sport specific)*	33^a^	33^a^	32^a^	26^b^	24^b^	31
Speed training*	25	30^a^	28	25^b^	21^b^	27
Speed endurance*	29	33^a^	28	26^b^	23^b^	28
Long endurance*	43^a^	43^a^	39	35^b^	30^b^	39
Interval/intermittent training*	41^a^	38^a^	35	31^b^	31^b^	35
Change of directions*	14	17^a^	17^a^	12^b^	10^b^	15
Others*	1	1	1	1	3^a^	1

Athletes could select multiple answers for all questions. Percentages within athlete classifications represent a “yes” answer relative to a “no” answer

*HIIT* high-intensity interval training, *INT* international, *NAT* national, *REC* recreational, *ST* state, *WC* world class

^a^Significantly higher

^b^Significantly lower

^*^Significant relationship with athlete classification (*χ*^2^), *p* < 0.05

## Discussion

These data represent the first global study reporting the knowledge and beliefs/attitudes of athletes (classified from Olympic to recreational level) regarding training disruptions and their practices during COVID-19 lockdown (March–June 2020). During lockdown, most athletes trained alone at their own homes, focusing on bodyweight-based and/or cardiovascular training promoting general fitness and health maintenance. Higher classification athletes were better able to maintain (e.g., resource access, including equipment and space) their pre-lockdown training specificity (e.g., plyometrics, technical skill, speed endurance, long endurance, and interval training). Training session frequency altered (from between five and seven sessions to four or fewer sessions per week), with shorter training sessions (≥ 60 to < 60 min) and lower sport-specific intensity (~ 38% reduction) for most athletes. Overall scores of knowledge and beliefs/attitudes related to training during lockdown were moderate and generally did not differ by athlete classification, except for recreational athletes, which were poor/moderate.

Training-related information is digitally and easily accessible for most athletes. Despite this, higher classification athletes appear to have learned “more” from their sporting experiences and networks, scoring higher than recreational athletes for knowledge and beliefs/attitudes about training (Table 3). Total scores were modest (range ~ 54–58%; moderate; see Table 3) across all surveyed athlete classifications, aside from recreational, suggesting that training-related evidence may not penetrate the knowledge and beliefs of all athletes. For example, less than half of the athletes (47%) indicated that endurance capacity could be maintained by doing high-intensity interval training (S2), despite demonstrated positive endurance training effects [29]. Similarly, only ~ 30% of athletes believed that < 4 weeks of lockdown would have little effect on their fitness levels (S3), a duration of ‘no training’ that may be tolerated without observing significant de-training effects [30, 31]. Athletes believed they needed “high training intensity” (71%; significantly more among international athletes) with “high training frequency” (88%) to maintain fitness level (S2), concurring with evidence that, to maintain or optimize endurance and strength performance effectively, training intensity must be “kept high” [30–33]. However, an important caveat is that, although strength levels can be retained for ≤ 3 weeks without resistance training, rates of strength decay may accelerate thereafter, i.e., ≥ 5 weeks [31]. The data and their agreement (or otherwise) with literature evidence should guide training prescriptions during periods of disruption (e.g., lockdown, illness, and injury), and it appears that palatable educational resources to this effect may be required to improve an apparent partial disconnect between the evidence–practitioner–athlete knowledge communication pathways.

During lockdown, athletes adapted to training with limited equipment and facilities [5] despite the likely low effectiveness of these approaches for optimal sports-specific training [3] and uncertain safety ramifications. In the current study, most athletes aimed to maintain/develop their general fitness/health and trained alone. Common training activities during lockdown were bodyweight exercises and cardiorespiratory training, probably because of the easy accessibility of these training modalities. Unfortunately for these athletes (in general), remote training (i.e., alone) reduced motivation (53%), a situation amplified by the lack of competition (58%), potentially leading to psychological issues, as reported elsewhere [34]. Such issues may be exacerbated by a lack of a “social facilitator” and encouragement [35] or simply missing interaction with team members [12]. These are substantial factors regarding social invitations for action (i.e., motivators) and athletes’ sport-related decision making [36]. Accordingly, higher classification athletes preferred training through cooperative/shared programming (e.g., athlete and coach input) and were more receptive to “remote training/coaching” (60%; highest amongst world-class athletes), evidently recognizing this (at least in part) as somewhat effective. Therefore, while we acknowledge the importance of maintaining “fitness” and physical qualities during lockdown, it is clear that mental and motivational aspects and training safety also warrant attention.

Training with sport specificity tailored towards key competition bouts/cycles requires inherent well-orchestrated variation in the key principles of training [4, 37, 38], which was evidently challenging during lockdown. Marked reductions in training frequency, duration, and intensity relative to before lockdown were reported, disproportionately affecting lower-level athletes compared with world-class and international athletes (Figs. 1 and 2). Irrespective of athlete classification, changes (i.e., reduced) in multiple training variables can compromise an athlete’s functional performance, especially if the training intensity is not maintained [30, 31, 33]. For example, among professional cyclists, changes in training volume and intensity distribution during a 7-week lockdown caused a large reduction in 5- and 20-min (maximal effort) cycling performance [39]. Total training volume decreased (− 34%), and the weekly volume of different standardized zones (i.e., zone 1 [low intensity] to zone 6 [high intensity]) was largely reduced (26–52%). Similarly, in a group of highly trained kayakers/canoeists, weekly training time and session duration reduced (− 28 and − 15%, respectively), albeit with no effects on the number of specific- and non-specific sessions [38]. Professional handball players saw marked reductions in training intensity (− 54%) and volume (− 90%) [40], as alluded to within the introduction regarding the susceptibility of team sports having their training demands severely compromised by lockdown. Athletes (predominantly collegiate level, based in the USA and from a variety of sports) experienced marked reductions in weekly training frequency (i.e., − 33% who trained for five to six sessions per week) and weekly time spent completing various training-related activities such as strength training (− 1.7 h), endurance (− 1.5 h), mobility (− 1.1 h), and sports specific (− 6.4 h) with lockdown [12]. Despite these evidently troubling lockdown-mediated training-related effects, several world records in athletics were broken during 2020–21 [44], raising questions about how certain athletes may have disproportionately benefited from lockdown, retaining a near-normal (perhaps augmented) training regimens during lockdown. Indeed, some elite athletes may have been able to execute training and recovery more effectively, facilitated in part by the reduced social, travel, and competition demands and preferential access to training equipment (e.g., weightlifting/strength training) through special arrangements (e.g., quarantine camp or training bubble) facilitating their normal (or augmented) training [3].

### Strengths, Limitations, and Future Directions

This study and the survey design have both strengths and limitations, and the presented data should be considered accordingly. A large (*n* = 12,526) sample of athletes was surveyed, providing a genuinely global (142 countries/territories across six continents) context for interpretation of the research questions. However, the results are time dependent given the cross-sectional nature of the study, and the data cannot be claimed to represent causative relationships. Indeed, the study explored lockdown at the beginning of the COVID-19 pandemic (March to June 2020), with most surveyed athletes (83%) experiencing a lockdown of 5–12 weeks during this period. Consequently, the potential for recall bias was present in some athletes; however, most questions were specific to athletes’ worst experiences during lockdown. Random sampling was adopted, which would avoid recruitment bias and improve internal validity, despite the well-reported weaknesses of online surveying [41]. Whether longer periods of lockdown and/or different time periods of restrictions may have yielded different data remains unknown and requires further investigation. Customized and bespoke survey questions were used, because existing surveys/questionnaires lacked the specificity or nuance required relative to the research questions being explored (i.e., an unprecedented pandemic). However, test–retest reliability for these questions were rated as good to excellent (Cronbach’s alpha 0.82–0.97). Furthermore, the knowledge and belief/attitude items were addressed in the first person, rather than the third person, to encourage athletes to respond instinctively to each question. Future studies may investigate similar challenges based on sex, sport “type,” geographical influence, and socio-economic and human development index factors. Additionally, a quantitative assessment of athletes’ physical qualities post-lockdown compared with robust pre-lockdown performance data benchmarking appears prudent to inform practice (i.e., reconditioning) and current/future policy in response to similar disruptions to athlete training.

## Conclusion

Higher classification athletes have superior knowledge and beliefs/attitudes regarding training, although these were ranked predominately as “moderate,” suggesting that training-related evidence may not penetrate to a “good” level in all athletes. COVID-19-mediated lockdown compromised nearly all aspects of effective training prescription and periodization (quantity and quality of training across intensity, duration, and frequency) in a manner disadvantageous to lower classification athletes. Lockdown elicited a change in athlete training behaviors, with more training alone and training to promote general health and well-being (i.e., remaining physically active) rather than with sport or discipline specificity, partly because of a lack of resource (e.g., space, equipment, facilities, and multidisciplinary support teams), with such access favoring higher classification athletes. Such training modifications reduced motivation in over half the athletes surveyed (and likely affected mental health in many more). The athlete–practitioner coaching/training interface saw the emergence of digitally mediated “remote”-based practices, which were best received by higher classification athletes. It would appear prudent to develop palatable athlete-centered (and practitioner) resources to improve their knowledge and beliefs/attitudes regarding training. Such upskilling would provide athletes with evidence to inform their training modifications in response to germane situations (e.g., COVID-related situations, injury, and illness). Sports organizations or teams should provide necessary resources to athletes, regardless of their classifications, by utilizing online learning and interaction platforms that offer free access to seminars and workshops. In this context, a specific approach to information delivery is required to target athletes across different classifications. The data suggest that stakeholders would benefit from policy and resources (including support) to facilitate remote training with their athletes. Furthermore, consideration of emerging technology (e.g., virtual reality) to diversify (improving motivation and engagement) lockdown-compatible training warrants discussion [42, 43]. Finally, these data and their context provide a clear rationale for careful consideration and prescription of appropriate sport-specific (re)conditioning upon return to “normal” training and/or competition to mitigate heightened injury risk [5, 8]. Holistically, stakeholders can use the data and discussion to develop policies, processes, and guidelines to facilitate training while keeping athletes safe and healthy (including mental health) during pandemic-related disruption to their training.

## Supplementary Information

Below is the link to the electronic supplementary material.

Supplementary file1 (DOCX 79 kb)

## Declarations

### Availability of data and material

All data are stored on institutional servers of the corresponding author and are available on reasonable request. All related survey questionnaires are presented in the main text or within the supplementary material.
