# Correction to: Training During the COVID-19 Lockdown: Knowledge, Beliefs, and Practices of 12,526 Athletes from 142 Countries and Six Continents

**DOI:** 10.1007/s40279-022-01776-y

**Published:** 2022-10-22

**Authors:** Jad Adrian Washif, Abdulaziz Farooq, Isabel Krug, David B. Pyne, Evert Verhagen, Lee Taylor, Del P. Wong, Iñigo Mujika, Cristina Cortis, Monoem Haddad, Omid Ahmadian, Mahmood Al Jufaili, Ramzi A. Al-Horani, Abdulla Saeed Al-Mohannadi, Asma Aloui, Achraf Ammar, Fitim Arifi, Abdul Rashid Aziz, Mikhail Batuev, Christopher Martyn Beaven, Ralph Beneke, Arben Bici, Pallawi Bishnoi, Lone Bogwasi, Daniel Bok, Omar Boukhris, Daniel Boullosa, Nicola Bragazzi, Joao Brito, Roxana Paola Palacios Cartagena, Anis Chaouachi, Stephen S. Cheung, Hamdi Chtourou, Germina Cosma, Tadej Debevec, Matthew D. DeLang, Alexandre Dellal, Gürhan Dönmez, Tarak Driss, Juan David Peña Duque, Cristiano Eirale, Mohamed Elloumi, Carl Foster, Emerson Franchini, Andrea Fusco, Olivier Galy, Paul B. Gastin, Nicholas Gill, Olivier Girard, Cvita Gregov, Shona Halson, Omar Hammouda, Ivana Hanzlíková, Bahar Hassanmirzaei, Thomas Haugen, Kim Hébert-Losier, Hussein Muñoz Helú, Tomás Herrera-Valenzuela, Florentina J. Hettinga, Louis Holtzhausen, Olivier Hue, Antonio Dello Iacono, Johanna K. Ihalainen, Carl James, Dina C. Janse van Rensburg, Saju Joseph, Karim Kamoun, Mehdi Khaled, Karim Khalladi, Kwang Joon Kim, Lian-Yee Kok, Lewis MacMillan, Leonardo Jose Mataruna-Dos-Santos, Ryo Matsunaga, Shpresa Memishi, Grégoire P. Millet, Imen Moussa-Chamari, Danladi Ibrahim Musa, Hoang Minh Thuan Nguyen, Pantelis T. Nikolaidis, Adam Owen, Johnny Padulo, Jeffrey Cayaban Pagaduan, Nirmala Panagodage Perera, Jorge Pérez-Gómez, Lervasen Pillay, Arporn Popa, Avishkar Pudasaini, Alireza Rabbani, Tandiyo Rahayu, Mohamed Romdhani, Paul Salamh, Abu-Sufian Sarkar, Andy Schillinger, Stephen Seiler, Heny Setyawati, Navina Shrestha, Fatona Suraya, Montassar Tabben, Khaled Trabelsi, Axel Urhausen, Maarit Valtonen, Johanna Weber, Rodney Whiteley, Adel Zrane, Yacine Zerguini, Piotr Zmijewski, Øyvind Sandbakk, Helmi Ben Saad, Karim Chamari

**Affiliations:** 1 Sports Performance Division, Institut Sukan Negara Malaysia (National Sports Institute of Malaysia), Kuala Lumpur, Malaysia; 2 Aspetar, Orthopaedic and Sports Medicine Hospital, FIFA Medical Centre of Excellence, Doha, Qatar; 3 Melbourne School of Psychological Sciences, The University of Melbourne, Melbourne, VIC Australia; 4 Research Institute for Sport and Exercise, University of Canberra, Canberra, ACT Australia; 5 Department of Public and Occupational Health, Amsterdam Collaboration on Health & Safety in Sports, Amsterdam Movement Sciences, Amsterdam UMC, Vrije Universiteit Amsterdam, Amsterdam, The Netherlands; 6 School of Sport, Exercise and Health Sciences, National Centre for Sport and Exercise Medicine (NCSEM), Loughborough University, Loughborough, UK; 7 Human Performance Research Centre, University of Technology Sydney, Sydney, Australia; 8 Sport & Exercise Discipline Group, Faculty of Health, University of Technology Sydney, Sydney, NSW Australia; 9 School of Nursing and Health Studies, The Open University of Hong Kong, Ho Man Tin, Hong Kong; 10 Department of Physiology, Faculty of Medicine and Nursing, University of the Basque Country, Leioa, Basque Country Spain; 11 Exercise Science Laboratory, Faculty of Medicine, School of Kinesiology, Universidad Finis Terrae, Santiago, Chile; 12 Department of Human Sciences, Society and Health, University of Cassino and Lazio Meridionale, Cassino, Italy; 13 Physical Education Department, College of Education, Qatar University, Doha, Qatar; 14 Medical Committee of Tehran Football Association, Tehran, Iran; 15 Emergency Medicine Department, Sultan Qaboos University Hospital, Alkhoudh, Oman; 16 Department of Exercise Science, Yarmouk University, Irbid, Jordan; 17 World Innovation Summit for Health (WISH), Qatar Foundation, Doha, Qatar; 18 Physical Activity, Sport & Health Research Unit (UR18JS01), National Sport Observatory, Tunis, Tunisia; 19 High Institute of Sport and Physical Education, University of Gafsa, Gafsa, Tunisia; 20 Institute of Sport Sciences, Otto-Von-Guericke University, 39104 Magdeburg, Germany; 21 Interdisciplinary Laboratory in Neurosciences, Physiology and Psychology: Physical Activity, Health and Learning (LINP2), UFR STAPS, UPL, Paris Nanterre University, Nanterre, France; 22 Physical Culture, Sports and Recreation, College Universi, Pristina, Kosovo; 23 Faculty of Physical Education and Sport, University of Tetova, Tetovo, North Macedonia; 24 Sport Science and Sport Medicine, Singapore Sport Institute, Sport Singapore, Singapore, Singapore; 25 Department of Sport, Exercise and Rehabilitation, Northumbria University, Newcastle upon Tyne, UK; 26 Division of Health, Engineering, Computing and Science, Te Huataki Waiora School of Health, University of Waikato, Tauranga, New Zealand; 27 Division of Medicine, Training and Health, Institute of Sport Science and Motology, Philipps University Marburg, Marburg, Germany; 28 Applied Motion Department, Institute of Sport Research, Sports University of Tirana, Tirana, Albania; 29 Physiotherapy Department, Minerva Punjab Academy and Football Club, Mohali, Punjab India; 30 Department of Orthopedics, Nyangabgwe Hospital, Francistown, Botswana; 31 Botswana Football Association Medical Committee, Gaborone, Botswana; 32 Faculty of Kinesiology, University of Zagreb, Zagreb, Croatia; 33 High Institute of Sport and Physical Education, University of Sfax, Sfax, Tunisia; 34 INISA, Federal University of Mato Grosso do Sul, Campo Grande, Brazil; 35 Sport and Exercise Science, James Cook University, Townsville, QLD Australia; 36 Laboratory for Industrial and Applied Mathematics (LIAM), Department of Mathematics and Statistics, York University, Toronto, ON M3J 1P3 Canada; 37 Portugal Football School, Portuguese Football Federation, Oeiras, Portugal; 38 Facultad de Ciencias del Deporte, Universidad de Extremadura, Cáceres, Spain; 39 Tunisian Research Laboratory, Sport Performance Optimisation, National Center of Medicine and Science in Sports (CNMSS), Tunis, Tunisia; 40 Sports Performance Research Institute New Zealand, AUT University, Auckland, New Zealand; 41 Department of Kinesiology, Brock University, St. Catharines, ON Canada; 42 Faculty of Physical Education and Sport, University of Craiova, Craiova, Romania; 43 Faculty of Sport, University of Ljubljana, Ljubljana, Slovenia; 44 Department of Automation, Biocybernetics and Robotics, Jozef Stefan Institute, Ljubljana, Slovenia; 45 Right to Dream Academy, Old Akrade, Ghana; 46 Sport Science and Research Department, Centre Orthopédique Santy, FIFA Medical Centre of Excellence, Lyon, France; 47 Laboratoire Interuniversitaire de Biologie de la Motricité (LIBM EA 7424), Claude Bernard University (Lyon 1), Lyon, France; 48 Department of Sports Medicine, Hacettepe University, Ankara, Turkey; 49 Al Hilal Football Club, Riyadh, Saudi Arabia; 50 Paris Saint Germain FC, Paris, France; 51 Health and Physical Education Department, Prince Sultan University, Riyadh, Kingdom of Saudi Arabia; 52 Department of Exercise and Sport Science, University of Wisconsin-La Crosse, La Crosse, WI USA; 53 Sport Department, School of Physical Education and Sport, University of São Paulo, São Paulo, Brazil; 54 Interdisciplinary Laboratory for Research in Education, EA 7483, University of New Caledonia, Avenue James Cook, 98800 Nouméa, New Caledonia; 55 Sport and Exercise Science, School of Allied Health, Human Services and Sport, La Trobe University, Melbourne, VIC Australia; 56 New Zealand All Blacks, New Zealand Rugby, Wellington, New Zealand; 57 School of Human Science (Exercise and Sport Science), The University of Western Australia, Perth, WA Australia; 58 School of Behavioural and Health Sciences, McAuley at Banyo, Australian Catholic University, Brisbane, QLD Australia; 59 Interdisciplinary Laboratory in Neurosciences, Physiology and Psychology: Physical Activity, Health and Learning (LINP2), UPL, UFR STAPS, Paris Nanterre University, Nanterre, France; 60 Research Laboratory, Molecular Bases of Human Pathology, Faculty of Medicine, LR19ES13, University of Sfax, Sfax, Tunisia; 61 Sports Medicine Research Center, Neuroscience Institute, Tehran University of Medical Sciences, Tehran, Iran; 62 Iran Football Medical Assessments and Rehabilitation Center, IFMARC, Tehran, Iran; 63 School of Health Sciences, Kristiania University College, Oslo, Norway; 64 Department of Economic-Administrative Sciences, Universidad Autónoma de Occidente, Los Mochis, Sinaloa México; 65 Department of Sport Science and Health, Universidad Santo Tomás, Santiago, Chile; 66 University of Santiago of Chile (USACH), Sciences of Physical Activity, Sports and Health School, Santiago, Chile; 67 Weil-Cornell Medical College in Qatar, Doha, Qatar; 68 Section Sports Medicine, Faculty of Health Sciences, University of Pretoria, Pretoria, South Africa; 69 Department of Exercise and Sports Science, University of the Free State, Bloemfontein, South Africa; 70 Laboratoire ACTES, UFR-STAPS, Université Des Antilles, Pointe à Pitre, France; 71 School of Health and Life Sciences, University of the West of Scotland, Hamilton, UK; 72 Faculty of Sport and Health Sciences, Biology of Physical Activity, University of Jyväskylä, Jyväskylä, Finland; 73 Medical Board Member, International Netball Federation, Manchester, UK; 74 High Performance Director, Sports Authority of India, Bangalore, India; 75 SEHA, Singapore, Singapore; 76 Department of Internal Medicine, Yonsei University College of Medicine, Seoul, South Korea; 77 Department of Sport Science, Tunku Abdul Rahman University College, Kuala Lumpur, Malaysia; 78 Sport Science Department, Fulham Football Club, Fulham, London, UK; 79 Centre for Trust, Peace and Social Relation, Coventry University, Coventry, UK; 80 Department of Sport Management, Faculty of Management, Canadian University of Dubai, Dubai, United Arab Emirates; 81 Programa Avancado de Cultura Contemporanea, Universidade Federal Do Rio de Janeiro, Rio de Janeiro, Brazil; 82 Antlers Sports Clinic, Kashima, Ibaraki Japan; 83 Department of Orthopedic Surgery, Tokyo Medical University, Tokyo, Japan; 84 Faculty of Physical Education, University of Tetovo, Tetovo, North Macedonia; 85 Institute of Sport Sciences, University of Lausanne, Lausanne, Switzerland; 86 Department of Human Kinetics and Health Education, Kogi State University, Anyigba, Nigeria; 87 Ho Chi Minh City University of Sport, Ho Chi Minh, Vietnam; 88 School of Health and Caring Sciences, University of West Attica, Attica, Greece; 89 University Claude Bernard Lyon 1, Lyon, France; 90 Seattle Sounders Football Club, Seattle, WA USA; 91 Department of Biomedical Sciences for Health, Università Degli Studi di Milano, Milan, Italy; 92 School of Health Sciences, College of Health and Medicine, University of Tasmania, Launceston, TAS Australia; 93 Sports Medicine, Australian Institute of Sport, Bruce, ACT Australia; 94 University of Canberra Research Institute for Sport and Exercise (UCRISE), University of Canberra, Bruce, ACT Australia; 95 Nuffield Department of Orthopaedics, Rheumatology and Musculoskeletal Sciences, University of Oxford, Oxford, UK; 96 Health, Economy, Motricity and Education (HEME) Research Group, Faculty of Sport Sciences, University of Extremadura, Cáceres, Spain; 97 University of Witwatersrand, Wits Institute for Sports Health, Johannesburg, South Africa; 98 Health and Sport Science Department, Educational Faculty, Mahasarakham University, Mahasarakham, Thailand; 99 Medical Department, All Nepal Football Association (ANFA), Lalitpur, Nepal; 100 Department of Exercise Physiology, College of Sport Sciences, University of Isfahan, Isfahan, Iran; 101 Faculty of Sport Science, Universitas Negeri Semarang, Semarang, Indonesia; 102 Krannert School of Physical Therapy, University of Indianapolis, Indianapolis, IN USA; 103 Bashundhara Kings, Nilphamari, Bangladesh; 104 Miskawaan Health Group, Bangkok, Thailand; 105 Department of Sports Science and Physical Education, University of Agder, Kristiansand, Norway; 106 Physiotherapy Department, BP Eyes Foundation CHEERS Hospital, Bhaktapur, Nepal; 107 Research Laboratory: Education, Motricity, Sport and Health, EM2S, LR19JS01, University of Sfax, Sfax, Tunisia; 108 Sports Clinic, Centre Hospitalier de Luxembourg, Clinique d‘Eich, Luxembourg, Luxembourg; 109 Luxembourg Institute of Research in Orthopedics, Sports Medicine and Science, Luxembourg, Luxembourg; 110 Human Motion, Orthopedics, Sports Medicine and Digital Methods, Luxembourg Institute of Health, Luxembourg, Luxembourg; 111 Research Institute for Olympic Sports, Jyvaskyla, Finland; 112 Institute for Sports Science, CAU of Kiel, Kiel, Germany; 113 Neurocognition and Action, University of Bielefeld, Bielefeld, Germany; 114 University of Queensland, Brisbane, QLD Australia; 115 Department of Physiology and Lung Function Testing, Faculty of Medicine of Sousse, University of Sousse, Sousse, Tunisia; 116 Faculty of Sciences of Bizerte, University of Carthage, Bizerte, Tunisia; 117 High Institute of Sports, Ksar Said, Tunis, Tunisia; 118 FIFA Medical Centre of Excellence Algiers, Algiers, Algeria; 119 Medical Committee, Confederation of African Football, Giza, Egypt; 120 Jozef Pilsudski University of Physical Education in Warsaw, Warsaw, Poland; 121 Centre for Elite Sports Research, Department of Neuromedicine and Movement Science, Norwegian, University of Science and Technology, Trondheim, Norway; 122 Laboratoire de Recherche “Insuffisance Cardiaque” (LR12SP09), Hôpital Farhat HACHED, Université de Sousse, Sousse, Tunisie; 123 Laboratoire de Physiologie, Faculté de Médicine de Sousse, Université de Sousse, Sousse, Tunisie

## Correction to: Sports Medicine (2021) 52:933–948 10.1007/s40279-021-01573-z

The affiliation of the author Lone Bogwasi, which previously read as:

Lone Bogwasi

Department of Orthopedics, Nyangabgwe Hospital, Francistown, Botswana

Botswana Football Association Medical Committee, Gaborone, Botswana

Now reads as:

Lone Bogwasi

Department of Orthopedics, Nyangabgwe Hospital, Francistown, Botswana

Botswana Football Association Medical Committee, Gaborone, Botswana

Section Sports Medicine, Faculty of Health Sciences, University of Pretoria, Pretoria, South Africa

The original article has been corrected.

