# Supplementary material for: Training During the COVID-19 Lockdown: Knowledge, Beliefs, and Practices of 12,526 Athletes from 142 Countries and Six Continents
Source: Sports Med. 2021 Oct 23;52(4):933–48. doi: 10.1007/s40279-021-01573-z (PMC8536915; doi:10.1007/s40279-021-01573-z)
Supplement: Supplementary file 1 — Supplementary file1 (DOCX 79 kb) [file 40279_2021_1573_MOESM1_ESM.docx]

**Electronic Supplementary Material**

**Journal:** Sports Medicine

**Title:** Training During the COVID-19 Lockdown: Knowledge, Beliefs, and Practices of 12,526 Athletes from 142 Countries and Six Continents

**Authors:** Washif JA^1 *^ (first author) …. Chamari K^2^ (last author)

**Affiliations:** ^1^Sports Performance Division, Institut Sukan Negara Malaysia (National Sports Institute of Malaysia), Kuala Lumpur, Malaysia; ^2^Aspetar, Orthopaedic and Sports Medicine Hospital, FIFA Medical Centre of Excellence, Doha, Qatar

***Contact:** [jad@isn.gov.my](mailto:jad@isn.gov.my)

**S1: Calculation of sample size.**

The sample size was calculated according to the following predictive equation (Whitley & Ball, 2003):


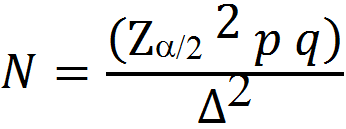


where N = number of needed athletes; Z_α/2_ = two-tailed normal deviate for type 1 error (Z_α/2_ =3.29 for 99.9% level of significance); q = 1-p; Δ = accuracy (=1.44%), and p = percentage of athletes who continued to train on a daily basis. According to Pillay et al. (2020), among the included 692 athletes, 422 (p = 0.61) continued to train on a daily basis. The sample size was therefore 12418 consecutive athletes. The assumption of 10% for missing or duplicate data gave a revised sample of 13798 athletes [13798 = 12418/ (1.00-0.10)].

**References**

1. Whitley E, Ball J. Statistics review 4: sample size calculations. Crit Care 2002;6:335-341.

2. Pillay L, Janse van Rensburg DC, Jansen van Rensburg A, et al. Nowhere to hide: the significant impact of coronavirus disease 2019 (COVID-19) measures on elite and semi-elite South African athletes. J Sci Med Sport 2020;23:670-679. <https://doi.org/10.1016/j.jsams.2020.05.016>

**S2:** Participants’ responses to training interruption and its consequences during COVID-19 lockdown (n = 12,495).

|  | **WC** | **INT** | **NAT** | **ST** | **REC** | **Overall** | **P ‡** |
| --- | --- | --- | --- | --- | --- | --- | --- |
|  | Correct or ‘positive’ answer, % | | | | | | |
| **K1:** Lockdown resulting in limited training can/has the potential to reduce my fitness level ^(C)^ | 68 | 68 | 68 | 69 | 62^b^ | 68 | 0.005 |
| **K2:** To maintain my fitness: I must keep my training volume (duration) high ^(F)^ | 12^b^ | 12 | 14 | 13 | 18^a^ | 13 | <0.001 |
| **K3:** To maintain my fitness: I must keep my training intensity high ^(C)^ | 72 | 74^a^ | 71 | 71 | 62^b^ | 71 | <0.001 |
| **K4:** If I decrease my training frequency (number of training sessions per week) by 30% or more, my fitness will decrease in a few weeks ^(C)^ | 87 | 89 | 87 | 88 | 85 | 88 | 0.079 |
| **K5:** Lockdown can/has reduced my technical skills ^(C)^ | 58 | 59 | 56 | 57 | 51^b^ | 57 | 0.002 |
| **K6:** Lockdown is a challenge for my mental health ^(C)^ | 56 | 56^b^ | 59 | 61 | 56 | 58 | <0.001 |
| During the lockdown, I can perform normal training as usual ^(NS)^ |  |  |  |  |  |  |  |
| **K7:** I think it is OKAY to not train at all during the lockdown ^(F)^ | 83^a^ | 82^a^ | 77 | 78 | 71^b^ | 79 | <0.001 |
| **K8:** During the lockdown, my aerobic fitness (endurance capacity) can be maintained by doing bodyweight-based High-Intensity Interval Training ^(C)^ | 48 | 49 | 47 | 47 | 44 | 47 | 0.089 |
| **K9:** During the lockdown, my pre-lockdown muscular strength can be trained / maintained by lifting light weights ^(F)^ | 32^a^ | 30 | 30 | 27^b^ | 28 | 30 | 0.004 |

‡ Using Chi-square test for independence; %, within athlete’s levels, represent ‘yes’ answer, relative to ‘no’ answer; ^a^, significantly higher; ^b^, significantly lower;

WC = world class, INT = international, NAT = national, ST = state, REC = recreational; (C) = correct statement, (F) = false, (NS) = non-scored, K = knowledge.

**S3:** Participants’ beliefs/attitudes related to training interruption during COVID-19 lockdown (n = 12,495).

|  | **WC** | **INT** | **NAT** | **ST** | **REC** | **Overall** | **P ‡** |
| --- | --- | --- | --- | --- | --- | --- | --- |
|  | ‘Positive’ answer, % | | | | | | |
| **B1:** Less than 4 weeks of lockdown will have little or no effect on my fitness levels ^(C)^ | 33^a^ | 30 | 28 | 29 | 32 | 30 | 0.002 |
| **B2:** Lockdown can make me mentally vulnerable ^(C)^ | 40^b^ | 42^b^ | 46 | 50^a^ | 45 | 45 | <0.001 |
| **B3:** I want to maintain my fitness during the lockdown period ^(C)^ | 87 | 87 | 83 | 86 | 78^b^ | 85 | <0.001 |
| My coach wants me to maintain my fitness during the lockdown period ^(NS)^ |  |  |  |  |  |  |  |
| **B4:** I think it is sufficient and effective to interact with my coach by correspondence during the lockdown period (e.g., video conferencing, messages, phone calls, others…) ^(C)^ | 65^a^ | 63 | 60 | 58 | 47^b^ | 60 | <0.001 |
| **B5:** I want(ed) to train during lockdown, but I don’t (did not) know how ^(F)^ | 61^a^ | 55 | 52 | 51 | 50^b^ | 54 | <0.001 |
| I have found video conferencing/tutorials online/TV for training with very little equipment needed (bodyweight) ^(NS)^ |  |  |  |  |  |  |  |
| I could train as I wished during lockdown ^(NS)^ |  |  |  |  |  |  |  |
| **B6:** Training during lockdown is often boring and less motivating ^(C)^ | 52 | 53 | 55 | 55 | 49 | 54 | 0.009 |
| During lockdown I feel/felt anxious ^(NS)^ |  |  |  |  |  |  |  |
| During the lockdown I am/was constantly scared to get infected by the COVID-19 virus ^(NS)^ |  |  |  |  |  |  |  |
| When in lockdown I think (I was thinking) that lockdown period may last longer than expected ^(NS)^ |  |  |  |  |  |  |  |
| **B7:** The absence of competition impacts(ed) on my training motivation ^(C)^ | 55 | 59 | 58 | 58 | 40^b^ | 57 | <0.001 |
| During lockdown, I feel/felt that I can/could do more training if racing against others ^(NS)^ |  |  |  |  |  |  |  |

‡ Using Chi-square test for independence; %, within athlete’s levels, represent ‘yes’ answer, relative to ‘no’ answer; ^a^ significantly higher; ^b^ significantly lower;

WC = world class, INT = international, NAT = national, ST = state, REC = recreational; (C) = correct statement, (F) = false, (NS) = non-scored, B = beliefs/attitudes.

**S4:** Participants’ knowledge as classified by athlete classification (n = 12,495).

|  | Strongly Agree (%) | Agree  (%) | Neutral  (%) | Disagree  (%) | Strongly Disagree (%) | Don’t Know (%) | *P* ^‡^ |
| --- | --- | --- | --- | --- | --- | --- | --- |
| **Q1. Lockdown resulting in limited training can/has the potential to reduce my fitness level** | | | | | | | |
| World class | 29 | 40 | 13 | 12^a^ | *5* | 2^a^ | <0.001 |
| International | *28* | 41 | 15 | 9 | 6 | 2 |  |
| National | 31^a^ | *38* | 13 | 9 | 6 | 3^a^ |  |
| State | 29 | 40 | 14 | 8 | 6 | 3 |  |
| Recreational | 26 | 37 | 15 | 10 | 9^a^ | 3 |  |
| Overall | 29 | 39 | 14 | 9 | 6 | 3 |  |
| **Q2. To maintain my fitness: I must keep my training volume (duration) high** | | | | | | | |
| World class | 30^a^ | 43 | 14 | 8 | 3^b^ | 2 | <0.001 |
| International | 28 | 46 | 13 | 8 | 5 | 2 |  |
| National | 26 | 44 | 14 | 8 | 7^a^ | 2 |  |
| State | 25 | 46 | 14 | 8 | 4^b^ | 2 |  |
| Recreational | 19^b^ | 44 | 16 | 9 | 10^a^ | 3 |  |
| Overall | 26 | 45 | 14 | 8 | 5 | 2 |  |
| **Q3. To maintain my fitness: I must keep my training intensity high** | | | | | | | |
| World class | 28 | 44 | 15 | 8^a^ | 3^b^ | 1 | <0.001 |
| International | 28 | 45 | 14 | 7 | 4 | 1 |  |
| National | 27 | 44 | 15 | 6 | 6^a^ | 2 |  |
| State | 26 | 46 | 17 | 7 | 3 | 2 |  |
| Recreational | 19^b^ | 44 | 19^a^ | 8 | 8^a^ | 2 |  |
| Overall | 27 | 45 | 16 | 7 | 4 | 2 |  |
| **Q4. If I decrease my training frequency (number of training sessions per week) by 30% or more, my fitness will decrease in a few weeks.** | | | | | | | |
| World class | 23 | 46 | 16 | 9 | 4 | 2 | 0.073 |
| International | 23 | 48 | 16 | 8 | 4 | 2 |  |
| National | 22 | 48 | 16 | 8 | **5** | 2 |  |
| State | 22 | 48 | 16 | 8 | 4 | 3 |  |
| Recreational | 21 | 47 | 15 | 9 | **6** | 2 |  |
| Overall | 22 | 48 | 16 | 8 | 5 | 2 |  |
| **Q5. Lockdown can/has reduced my technical skills** | | | | | | | |
| World class | 24^a^ | 34 | 16^b^ | 18^a^ | 6 | 2 | <0.001 |
| International | 22 | 36 | 18 | 15 | 6 | 3 |  |
| National | 21 | 35 | 19 | 15 | 7 | 3 |  |
| State | 21 | 36 | 19 | 16 | 6 | 2^b^ |  |
| Recreational | 18^b^ | 34 | 18 | 17 | 10^a^ | 3 |  |
| Overall | 21 | 36 | 18 | 16 | 7 | 3 |  |
| **Q6. Lockdown is a challenge for my mental health** | | | | | | | |
| World class | 22^b^ | 34 | 17 | 16^a^ | 9 | 2^b^ | <0.001 |
| International | 25 | 32 | 18 | 16^a^ | 7 | 3 |  |
| National | 26 | 34 | 17 | 12^b^ | 8 | 3 |  |
| State | 27 | 34 | 18 | 13^b^ | 7 | 2^b^ |  |
| Recreational | 25 | 32 | 19 | 11^b^ | 9 | 4^a^ |  |
| Overall | 25 | 33 | 18 | 14 | 8 | 2 |  |
| **Q7. During the lockdown period, I can perform normal training as usual** | | | | | | | |
| World class | 4 | 16 | 14^b^ | 36 | 30 ^a^ | 0^b^ | <0.001 |
| International | 3^b^ | 16 | 17 | 39 | 25 | 1 |  |
| National | 5 | 16 | 17 | 37 | 25 | 1 |  |
| State | 5 | 17 | 17 | 36 | 23 | 1 |  |
| Recreational | 5 | 20^a^ | 17 | 32^b^ | 23 | 3^a^ |  |
| Overall | 5 | 16 | 17 | 37 | 25 | 1 |  |
| **Q8. I think it is OKAY to not train at all during the lockdown period** | | | | | | | |
| World class | 2 | 6^b^ | 8^b^ | 27 | 56^a^ | 1 | <0.001 |
| International | 2 | 8 | 8^b^ | 30 | 53 | 0^b^ |  |
| National | 2 | 10^a^ | 10 | 28 | 50 | 1 |  |
| State | 3 | 9 | 10 | 29 | 49 | 1 |  |
| Recreational | 2 | 13^a^ | 12^a^ | 27 | 45^b^ | 2 |  |
| Overall | 2 | 9 | 9 | 28 | 51 | 1 |  |
| **Q9. During the lockdown period, my aerobic fitness (endurance capacity) can be maintained by doing bodyweight-based High-Intensity Interval Training** | | | | | | | |
| World class | 10 | 39 | 23 | 18 | 7 | 4 | <0.001 |
| International | 10 | 39 | 24 | 18 | 6^b^ | 3^b^ |  |
| National | 9 | 37 | 25 | 18 | 7 | 4 |  |
| State | 10 | 37 | 24 | 18 | 6 | 5 |  |
| Recreational | 8 | 37 | 21 | 17 | 9^a^ | 8^a^ |  |
| Overall | 10 | 38 | 24 | 18 | 7 | 4 |  |
| **Q10. During the lockdown period, my pre-lockdown muscular strength can be trained / maintained by lifting light weights** | | | | | | | |
| World class | 7 | 37 | 21 | 22 | 10 | 3 | <0.001 |
| International | 6 | 38 | 23 | 22 | 8 | 3 |  |
| National | 7 | 38 | 23 | 21 | 9 | 3 |  |
| State | 7 | 40 | 22 | 20 | 8^b^ | 3 |  |
| Recreational | 6 | 40 | 21 | 19 | 10 | 5^a^ |  |
| Overall | 7 | 38 | 22 | 21 | 9 | 3 |  |

‡ Using Chi-square test for independence; %, within athlete’s levels, represent ‘yes’ answer, relative to ‘no’ answer; ^a^ significantly higher; ^b^ significantly lower;

**N.B.** – 36% agreed and 21% strongly agreed that movement restrictions could reduce technical skill (Q5); world class and recreational athletes had higher (24%) and lower (18%) scores for strongly agree, respectively (p<0.05). 33% agreed and 25% strongly agreed that movement restrictions were mentally challenging (Q6); less (strongly agreed) among world-class athletes (p<0.05). Moreover, 51% strongly disagreed for “OKAY to not train at all” statement (Q8) during movement restrictions; here, world-class had highest score while recreational athletes had lowest score (p<0.05). *Other details are described in the main text.*

**S5:** Participants’ belief/attitude/fact/feeling as classified by athlete classification (n = 12,495).

|  | Strongly Agree (%) | Agree (%) | Neutral (%) | Disagree (%) | Strongly Disagree (%) | Don’t Know  (%) | *P* ^‡^ |
| --- | --- | --- | --- | --- | --- | --- | --- |
| **Q1. Less than 4 weeks of lockdown will have little or no effect on my fitness levels** | | | | | | | |
| World class | 6^a^ | 27 | 20 | 35 | 11 | 2 | <0.001 |
| International | 4 | 26 | 22 | 35 | 11 | 2 |  |
| National | 5 | 24 | 21 | 36 | 13 | 2 |  |
| State | 5 | 24 | 21 | 38 | 10 | 2 |  |
| Recreational | 6 | 27 | 21 | 30^b^ | 14 | 3 |  |
| Overall | 5 | 25 | 21 | 36 | 12 | 2 |  |
| **Q2. Lockdown can make me mentally vulnerable** | | | | | | | |
| World class | 10^b^ | 30^b^ | 22 | 25^a^ | 11 | 1 | <0.001 |
| International | 11 | 31 | 23 | 25^a^ | 9 | 1 |  |
| National | 12 | 34 | 21 | 23 | 8 | 2 |  |
| State | 13 | 37^a^ | 21 | 20^b^ | 8 | 2 |  |
| Recreational | 14 | 32 | 22 | 21 | 8 | 4^a^ |  |
| Overall | 12 | 34 | 22 | 23 | 8 | 2 |  |
| **Q3. I want to maintain my fitness during the lockdown period** | | | | | | | |
| World class | 51^a^ | 36 | 5 | 2 | 3^b^ | 3^b^ | <0.001 |
| International | 51^a^ | 37 | 4^b^ | 1 | 3^b^ | 4 |  |
| National | 45^b^ | 39 | 5 | 2 | 5^a^ | 5^a^ |  |
| State | 47 | 39 | 6 | 2 | 4 | 3^b^ |  |
| Recreational | 39^b^ | 41 | 6 | 1 | 6^a^ | 8^a^ |  |
| Overall | 47 | 38 | 5 | 1 | 4 | 4 |  |
| **Q4. My coach wants me to maintain my fitness during the lockdown period** | | | | | | | |
| World class | 42^a^ | 40 | 9 | 3 | 4^b^ | 4^b^ | <0.001 |
| International | 44^a^ | 37 | 8^b^ | 3 | 4^b^ | 5^b^ |  |
| National | 37 | 38 | 10 | 3 | 5 | 7^b^ |  |
| State | 33^b^ | 38 | 14^a^ | 3 | 4^a^ | 9^a^ |  |
| Recreational | 21^b^ | 38 | 16^a^ | 4 | 7^a^ | 14^a^ |  |
| Overall | 37 | 38 | 11 | 3 | 5 | 7 |  |
| **Q5. I think it is sufficient and effective to interact with my coach by correspondence during the lockdown period (e.g., video-conferencing, messages, phone calls, others…)** | | | | | | | |
| World class | 25^a^ | 40 | 15^b^ | 12 | 5 | 3^b^ | <0.001 |
| International | 23 | 39 | 18 | 11 | 5 | 3^b^ |  |
| National | 22 | 39 | 17 | 12 | 6^a^ | 4^b^ |  |
| State | 20^b^ | 38 | 19 | 12 | 4^b^ | 6^a^ |  |
| Recreational | 15^b^ | 33^b^ | 21^a^ | 12 | 7 | 12^a^ |  |
| Overall | 22 | 39 | 18 | 12 | 5 | 5 |  |
| **Q6. I want(ed) to train during lockdown, but I don’t (did not) know how** | | | | | | | |
| World class | 4^b^ | 13^b^ | 19 | 37 | 25^a^ | 2 | <0.001 |
| International | 5^b^ | 17 | 22 | 36 | 19 | 1^b^ |  |
| National | 6 | 18 | 21 | 36 | 17^b^ | 2 |  |
| State | 7^a^ | 19 | 22 | 34 | 17^b^ | 2 |  |
| Recreational | 6 | 20 | 20 | 31^b^ | 20 | 2 |  |
| Overall | 6 | 18 | 21 | 35 | 19 | 2 |  |
| **Q7. I have found video-conferencing/tutorials online/TV for training with very little equipment needed (bodyweight). I think they can be helpful in maintaining athletes’ fitness.** | | | | | | | |
| World class | 15 | 46 | 20^a^ | 8 | 7 | 4 | <0.001 |
| International | 15 | 49^a^ | 18 | 8 | 7 | 3 |  |
| National | 17 | 46 | 18 | 8 | 7 | 4 |  |
| State | 19^a^ | 46 | 18 | 8 | 6 | 3 |  |
| Recreational | 17 | 43 | 14^b^ | 9 | 9^a^ | 7^a^ |  |
| Overall | 17 | 47 | 18 | 8 | 7 | 4 |  |
| **Q8. I could train as I wished during lockdown** | | | | | | | |
| World class | 9 | 25 | 16^b^ | 31 | 19^a^ | 1 | 0.021 |
| International | 8 | 25 | 20 | 31 | 16 | 1 |  |
| National | 8 | 24 | 19 | 31 | 17 | 1 |  |
| State | 9 | 23 | 20 | 31 | 15 | 1 |  |
| Recreational | 8 | 25 | 18 | 30 | 17 | 2^a^ |  |
| Overall | 8 | 24 | 19 | 31 | 17 | 1 |  |
| **Q9. Training during lockdown is often boring and less motivating** | | | | | | | |
| World class | 15^b^ | 37 | 19 | 20^a^ | 7 | 2^b^ | <0.001 |
| International | 19 | 34^b^ | 19 | 18 | 8 | 3 |  |
| National | 19^a^ | 36 | 17 | 15^b^ | 9 | 3 |  |
| State | 17 | 39^a^ | 18 | 16 | 8 | 3 |  |
| Recreational | 15 | 35 | 18 | 17 | 11^a^ | 4^a^ |  |
| Overall | 18 | 36 | 18 | 17 | 9 | 3 |  |
| **Q10. During lockdown I feel/felt anxious** | | | | | | | |
| World class | 10^b^ | 31 | 21 | 24^a^ | 13^a^ | 2 | <0.001 |
| International | 11^b^ | 32 | 23 | 22 | 11 | 3 |  |
| National | 13 | 33 | 21 | 20 | 11 | 3 |  |
| State | 15^a^ | 34 | 21 | 19 | 10^b^ | 2 |  |
| Recreational | 13 | 29 | 22 | 18 | 14^a^ | 4^a^ |  |
| Overall | 13 | 33 | 21 | 20 | 11 | 3 |  |
| **Q11. During the lockdown I am/was constantly scared to get infected by the COVID-19 virus** | | | | | | | |
| World class | 9 ^b^ | 21^b^ | 20 | 30^a^ | 19^a^ | 1^b^ | <0.001 |
| International | 12 | 25 | 22 | 26 | 14 | 2 |  |
| National | 11 | 26 | 22 | 25 | 15 | 2 |  |
| State | 11 | 27^a^ | 22 | 2 ^b^ | 15 | 2 |  |
| Recreational | 12 | 26 | 23 | 23 | 14 | 3 |  |
| Overall | 11 | 26 | 22 | 25 | 15 | 2 |  |
| **Q12. When in lockdown I think (I was thinking) that lockdown period may last longer than expected** | | | | | | | |
| World class | 18^b^ | 44 | 16^a^ | 13^a^ | 5 | 3 | <0.001 |
| International | 22 | 44 | 15 | 10 | 5 | 3 |  |
| National | 22 | 45 | 14 | 10 | 6 | 3 |  |
| State | 24^a^ | 44 | 13^b^ | 11 | 5 | 3 |  |
| Recreational | 22 | 40 | 14 | 12 | 8^a^ | 4 |  |
| Overall | 22 | 44 | 14 | 11 | 6 | 3 |  |
| **Q13. The absence of competition impacts(ed) on my training motivation** | | | | | | | |
| World class | 22 | 34 | 16 | 19^a^ | 9 | 1^b^ | <0.001 |
| International | 22 | 37 | 16 | 16 | 7 | 2 |  |
| National | 23 | 36 | 15 | 15 | 8 | 3 |  |
| State | 22 | 37 | 15 | 16 | 8 | 2 |  |
| Recreational | 13^b^ | 28^b^ | 24^a^ | 18 | 12^a^ | 5^a^ |  |
| Overall | 22 | 36 | 16 | 16 | 8 | 2 |  |
| **Q14. During lockdown, I feel/felt that I can/could do more training if racing against others. Training by myself without partners/opponents reduced my training load (volume/intensity)** | | | | | | | |
| World class | 12^b^ | 38 | 20 | 20^a^ | 7 | 3 | <0.001 |
| International | 16 | 38 | 20 | 15 | 8 | 3 |  |
| National | 17 | 37 | 19 | 15 | 9 | 4 |  |
| State | 17 | 38 | 19 | 15 | 8 | 3 |  |
| Recreational | 15 | 32^b^ | 21 | 18 | 10 | 4 |  |
| Overall | 16 | 37 | 19 | 16 | 8 | 3 |  |

‡ Using Chi-square test for independence; %, within athlete’s levels, represent ‘yes’ answer, relative to ‘no’ answer; ^a^ significantly higher; ^b^ significantly lower;

**N.B.** – A minority (36%) of athletes disagreed that 4 weeks of movement restrictions have little impact on fitness levels (Q1), less among recreational athletes (p<0.05). Similarly, 34% athletes agreed that movement restrictions can make an athlete mentally vulnerable (Q2); less among world-class and higher among state athletes (p<0.05). 37% of athletes strongly agreed that their coaches wanted them to maintain fitness during movement restrictions (Q4); (more world-class- and international-athletes indicated: strongly agree (p<0.05). 35% athletes disagreed to the statement of “I want to train during movement restrictions but I don’t know how” (less recreational athletes indicated: disagree (Q6), p<0.05). Moreover, 36% of athletes agreed that training during movement restrictions is often boring and less motivating (Q9) (less among international- (p<0.05) but more among state-athletes (p<0.05). Most athletes agreed (44%) and strongly agreed (22%) that the upcoming period of movement restrictions may last longer than expected (Q12). 36% athletes agreed and 22% strongly disagreed that the absence of competition impacted their training motivation (Q13) (p<0.05). *Other details are described in the main text.*
